# Time resolution dependence of information measures for spiking neurons: scaling and universality

**DOI:** 10.3389/fncom.2015.00105

**Published:** 2015-08-28

**Authors:** Sarah E. Marzen, Michael R. DeWeese, James P. Crutchfield

**Affiliations:** ^1^Department of Physics, University of California, BerkeleyBerkeley, CA, USA; ^2^Helen Wills Neuroscience Institute and Redwood Center for Theoretical Neuroscience, University of California, BerkeleyBerkeley, CA, USA; ^3^Complexity Sciences Center and Department of Physics, University of California, DavisDavis, CA, USA

**Keywords:** statistical complexity, excess entropy, entropy rate, renewal process, alternating renewal process, integrate and fire neuron, leaky integrate and fire neuron, quadratic integrate and fire neuron, 05.45.Tp 02.50.Ey 87.10.Vg 87.19.ll 87.19.lo 87.19.ls

## Abstract

The mutual information between stimulus and spike-train response is commonly used to monitor neural coding efficiency, but neuronal computation broadly conceived requires more refined and targeted information measures of input-output joint processes. A first step toward that larger goal is to develop information measures for individual output processes, including information generation (entropy rate), stored information (statistical complexity), predictable information (excess entropy), and active information accumulation (bound information rate). We calculate these for spike trains generated by a variety of noise-driven integrate-and-fire neurons as a function of time resolution and for alternating renewal processes. We show that their time-resolution dependence reveals coarse-grained structural properties of interspike interval statistics; e.g., τ-entropy rates that diverge less quickly than the firing rate indicated by interspike interval correlations. We also find evidence that the excess entropy and regularized statistical complexity of different types of integrate-and-fire neurons are universal in the continuous-time limit in the sense that they do not depend on mechanism details. This suggests a surprising simplicity in the spike trains generated by these model neurons. Interestingly, neurons with gamma-distributed ISIs and neurons whose spike trains are alternating renewal processes do not fall into the same universality class. These results lead to two conclusions. First, the dependence of information measures on time resolution reveals mechanistic details about spike train generation. Second, information measures can be used as model selection tools for analyzing spike train processes.

## 1. Introduction

Despite a half century of concerted effort (Mackay and McCulloch, 1952), neuroscientists continue to debate the relevant timescales of neuronal communication as well as the basic coding schemes at work in the cortex, even in early sensory processing regions of the brain thought to be dominated by feedforward pathways (Softky and Koch, 1993; Bell et al., 1995; Shadlen and Newsome, 1995; Stevens and Zador, 1998; Destexhe et al., 2003; DeWeese and Zador, 2006; Jacobs et al., 2009; Koepsell et al., 2010; London et al., 2010). For example, the apparent variability of neural responses to repeated presentations of sensory stimuli has led many to conclude that the brain must average across tens or hundreds of milliseconds or across large populations of neurons to extract a meaningful signal (Shadlen and Newsome, 1998). Whereas, reports of reliable responses suggest shorter relevant timescales and more nuanced coding schemes (Berry et al., 1997; Reinagel and Reid, 2000; DeWeese et al., 2003). In fact, there is evidence for different characteristic timescales for neural coding in different primary sensory regions of the cortex (Yang and Zador, 2012). In addition to questions about the relevant timescales of neural communication, there has been an ongoing debate regarding the magnitude and importance of correlations among the spiking responses of neural populations (Meister et al., 1995; Nirenberg et al., 2001; Averbeck et al., 2006; Schneidman et al., 2003, 2006).

Most studies of neural coding focus on the relationship between a sensory stimulus and the neural response. Others consider the relationship between the neural response and the animal's behavioral response (Britten et al., 1996), the relationship between pairs or groups of neurons at different stages of processing (Linsker, 1989; Dan et al., 1996), or the variability of neural responses themselves without regard to other variables (Schneidman et al., 2006). Complementing the latter studies, we are interested in quantifying the randomness and predictability of neural responses without reference to stimulus. We consider the variability of a given neuron's activity at one time and how this is related to the same neuron's activity at other times in the future and the past.

Along these lines, information theory (Shannon, 1948; Cover and Thomas, 2006) provides an insightful and rich toolset for interpreting neural data and for formulating theories of communication and computation in the nervous system (Rieke et al., 1999). In particular, Shannon's mutual information has developed into a powerful probe that quantifies the amount of information about a sensory stimulus encoded by neural activity (Mackay and McCulloch, 1952; Barlow, 1961; Stein, 1967; Laughlin, 1981; Sakitt and Barlow, 1982; Srinivasan et al., 1982; Linsker, 1989; Bialek et al., 1991; Theunissen and Miller, 1991; Atick, 1992; Rieke et al., 1999). Similarly, the Shannon entropy has been used to quantify the variability of the resulting spike-train response. In contrast to these standard stimulus- and response-averaged quantities, a host of other information-theoretic measures have been applied in neuroscience, such as the Fisher information (Cover and Thomas, 2006) and various measures of the information gained per observation (DeWeese and Meister, 1999; Butts and Goldman, 2006).

We take an approach that complements more familiar informational analyses. First, we consider “output-only” processes, since their analysis is a theoretical prerequisite to understanding information in the stimulus-response paradigm. Second, we analyze rates of informational divergence, not only nondivergent components. Indeed, we show that divergences, rather than being a kind of mathematical failure, are important and revealing features of information processing in spike trains.

We are particularly interested in the information content of neural spiking on fine timescales. How is information encoded in spike timing and, more specifically, in interspike intervals? In this regime, the critical questions turn on determining the kind of information encoded and the required “accuracy” of individual spike timing to support it. At present, unfortunately, characterizing communication at submillisecond time scales and below remains computationally and theoretically challenging.

Practically, a spike train is converted into a binary sequence for analysis by choosing a time bin size and counting the number of spikes in successive time bins. Notwithstanding Strong et al. (1998) and Nemenman et al. (2008), there are few studies of how estimates of communication properties change as a function of time bin size, though there are examples of both short (Panzeri et al., 1999) and long (DeWeese, 1996; Strong et al., 1998) time expansions. Said most plainly, it is difficult to directly calculate the most basic quantities—e.g., communication rates between stimulus and spike-train response—in the submillisecond regime, despite progress on undersampling (Treves and Panzeri, 1995; Nemenman et al., 2004; Archer et al., 2012). Beyond the practical, the challenges are also conceptual. For example, given that a stochastic process' entropy rate diverges in a process-characteristic fashion for small time discretizations (Gaspard and Wang, 1993), measures of communication efficacy require careful interpretation in this limit.

Compounding the need for better theoretical tools, measurement techniques will soon amass enough data to allow serious study of neuronal communication at fine time resolutions and across large populations (Alivisatos et al., 2012). In this happy circumstance, we will need guideposts for how information measures of neuronal communication vary with time resolution so that we can properly interpret the empirical findings and refine the design of nanoscale probes.

Many single-neuron models generate neural spike trains that are renewal processes (Gerstner and Kistler, 2002). Starting from this observation, we use recent results (Marzen and Crutchfield, 2015) to determine how information measures scale in the small time-resolution limit. This is exactly the regime where numerical methods are most likely to fail due to undersampling and, thus, where analytic formulae are most useful. We also extend the previous analyses to structurally more complex, alternating renewal processes and analyze the time-resolution scaling of their information measures. This yields important clues as to which scaling results apply more generally. We then show that, across several standard neuronal models, the information measures are universal in the sense that their scaling does not depend on the details of spike-generation mechanisms.

Several information measures we consider are already common fixtures in theoretical neuroscience, such as Shannon's source entropy rate (Strong et al., 1998; Nemenman et al., 2008). Others have appeared at least once, such as the finite-time excess entropy (or predictable information) (Bialek et al., 2001; Crutchfield and Feldman, 2003) and statistical complexity (Haslinger et al., 2010). And others have not yet been applied, such as the bound information (Abdallah and Plumbley, 2009, 2012; James et al., 2011, 2014).

The development proceeds as follows. Section 2 reviews notation and definitions. To investigate the dependence of causal information measures on time resolution, Section 3 studies a class of renewal processes motivated by their wide use in describing neuronal behavior. Section 4 then explores the time-resolution scaling of information measures of alternating renewal processes, identifying those scalings likely to hold generally. Section 5 evaluates continuous-time limits of these information measures for common single-neuron models. This reveals a new kind of universality in which the information measures' scaling is independent of detailed spiking mechanisms. Taken altogether, the analyses provide intuition and motivation for several of the rarely-used, but key informational quantities. For example, the informational signatures of integrate-and-fire model neurons differ from both simpler, gamma-distributed processes and more complex, compound renewal processes. Finally, Section 6 summarizes the results, giving a view to future directions and mathematical and empirical challenges.

## 2. Background

We can only briefly review the relevant physics of information. Much of the phrasing is taken directly from background presented in Marzen and Crutchfield (2014, 2015).

Let us first recall the causal state definitions (Shalizi and Crutchfield, 2001) and information measures of discrete-time, discrete-state processes introduced in Crutchfield et al. (2009), James et al. (2011). The main object of study is a process P: the list of all of a system's behaviors or realizations {…*x*_−2_, *x*_−1_, *x*_0_, *x*_1_, …} and their probabilities, specified by the joint distribution Pr(…*X*_−2_, *X*_−1_, *X*_0_, *X*_1_, …). We denote a contiguous chain of random variables as *X*_0:*L*_ = *X*_0_*X*_1_⋯*X*_*L*−1_. We assume the process is ergodic and stationary—Pr(*X*_0:*L*_) = Pr(*X*_*t*:*L*+*t*_) for all *t* ∈ ℤ —and the measurement symbols range over a finite alphabet: *x* ∈ A. In this setting, the *present X*_0_ is the random variable measured at *t* = 0, the *past* is the chain *X*_:0_ = …*X*_−2_*X*_−1_ leading up the present, and the *future* is the chain following the present *X*_1:_ = *X*_1_*X*_2_⋯ (We suppress the infinite index in these).

As the Introduction noted, many information-theoretic studies of neural spike trains concern input-output information measures that characterize stimulus-response properties; e.g., the mutual information between stimulus and resulting spike train. In the absence of stimulus or even with a non-trivial stimulus, we can still study neural activity from an information-theoretic point of view using “output-only” information measures that quantify intrinsic properties of neural activity alone:

- How random is it? The *entropy rate h*_μ_ = *H*[*X*_0_|*X*_:0_], which is the entropy in the present observation conditioned on all past observations (Cover and Thomas, 2006).
- What must be remembered about the past to optimally predict the future? The *causal states* S^+^, which are groupings of pasts that lead to the same probability distribution over future trajectories (Crutchfield and Young, 1989; Shalizi and Crutchfield, 2001).
- How much memory is required to store the causal states? The *statistical complexity* $C_{\mu }=H\left[S^{+}\right]$, or the entropy of the causal states (Crutchfield and Young, 1989).
- How much of the future is predictable from the past? The *excess entropy* **E** = *I*[*X*_:0_; *X*_0:_], which is the mutual information between the past and the future (Crutchfield and Feldman, 2003).
- How much of the generated information (*h*_μ_) is relevant to predicting the future? The *bound information b*_μ_ = *I*[*X*_0_; *X*_1:_|*X*_:0_], which is the mutual information between the present and future observations conditioned on all past observations (Abdallah and Plumbley, 2009; James et al., 2011).
- How much of the generated information is useless—neither affects future behavior nor contains information about the past? The *ephemeral information r*_μ_ = *H*[*X*_0_|*X*_:0_, *X*_1:_], which is the entropy in the present observation conditioned on all past and future observations (Verdú and Weissman, 2006; James et al., 2011).

**Figure 1:**
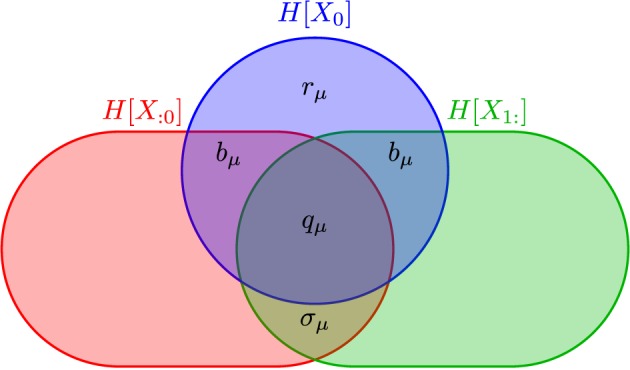
**Information diagram illustrating the anatomy of the information *H*[*X*_0_] in a process' single observation *X*_0_ in the context of its past *X*_:0_ and its future *X*_1:_**. Although the past entropy *H*[*X*_:0_] and the future entropy *H*[*X*_1:_] typically are infinite, space precludes depicting them as such. They do scale in a controlled way, however: *H*[*X*_ − ℓ:0_] ∝ *h*_μ_ℓ and *H*[*X*_1:ℓ_] ∝ *h*_μ_ℓ. The two atoms labeled *b*_μ_ are the same, since we consider only stationary processes. (After James et al., 2011, with permission.)

The *information diagram* of Figure 1 illustrates the relationship between *h*_μ_, *r*_μ_, *b*_μ_, and **E**. When we change the time discretization Δ*t*, our interpretation and definitions change somewhat, as we describe in Section 3.

Shannon's various information quantities—entropy, conditional entropy, mutual information, and the like—when applied to time series are functions of the joint distributions Pr(*X*_0:*L*_). Importantly, for a given set of random variables they define an algebra of *atoms* out of which information measures are composed (Yeung, 2008). James et al. (2011) used this to show that the past and future partition the single-measurement entropy *H*(*X*_0_) into the measure-theoretic atoms of Figure 1. These include those—*r*_μ_ and *b*_μ_—already mentioned and the *enigmatic information*:

$$q_{\mu }=I[X_{0};X_{:0};X_{1:}]\;,$$

which is the co-information between past, present, and future. One can also consider the amount of predictable information not captured by the present:

$$\sigma_{\mu }=I[X_{:0};X_{1:}|X_{0}].$$

which is the *elusive information* (Ara et al., 2015). It measures the amount of past-future correlation not contained in the present. It is nonzero if the process has “hidden states” and is therefore quite sensitive to how the state space is “observed” or coarse-grained.

The total information in the future predictable from the past (or vice versa)—the excess entropy—decomposes into particular atoms:

$$E=b_{\mu }+\;\sigma_{\mu }+{\text{ q}}_{\mu }.$$

The process's Shannon entropy rate *h*_μ_ is also a sum of atoms:

$$h_{\mu }=r_{\mu }+b_{\mu }\;.$$

This tells us that a portion of the information (*h*_μ_) a process spontaneously generates is thrown away (*r*_μ_) and a portion is actively stored (*b*_μ_). Putting these observations together gives the information anatomy of a single measurement *X*_0_:

(1) $$H[X_{0}]=q_{\mu }+2b_{\mu }+r_{\mu }\;.$$

Although these measures were originally defined for stationary processes, they easily carry over to a nonstationary process of finite Markov order.

Calculating these information measures in closed-form given a model requires finding the ϵ*-machine*, which is constructed from causal states. Forward-time causal states S^+^ are minimal sufficient statistics for predicting a process's future (Crutchfield and Young, 1989; Shalizi and Crutchfield, 2001). This follows from their definition—a *causal state* σ^+^ ∈ S^+^ is a sets of pasts grouped by the equivalence relation ~^+^:

(2) $$\begin{array}{l} x_{:0}\sim^{+}x_{:0}^{\prime }\\ \;\Leftrightarrow \text{Pr}(X_{0:}|X_{:0}=x_{:0})=\text{Pr}(X_{0:}|X_{:0}=x_{:0}^{\prime })\;. \end{array}$$

So, S^+^ is a set of classes—a coarse-graining of the uncountably infinite set of all pasts. At time *t*, we have the random variable $S_{t}^{+}$ that takes values σ^+^ ∈ S^+^ and describes the *causal-state process* $\ldots ,S_{-1}^{+},S_{0}^{+},S_{1}^{+},\ldots$. $S_{t}^{+}$ is a partition of pasts *X*_:*t*_ that, according to the indexing convention, does not include the present observation *X*_*t*_. In addition to the set of pasts leading to it, a causal state $\sigma_{t}^{+}$ has an associated *future morph*—the conditional measure $\text{Pr}\left(X_{t:}|\sigma_{t}^{+}\right)$ of futures that can be generated from it. Moreover, each state $\sigma_{t}^{+}$ inherits a probability $\pi \left(\sigma_{t}^{+}\right)$ from the process's measure over pasts Pr(*X*_:*t*_). The forward-time *statistical complexity* is then the Shannon entropy of the state distribution $\pi \left(\sigma_{t}^{+}\right)$ (Crutchfield and Young, 1989): $C_{\mu }^{+}=H\left[S_{0}^{+}\right]$. A generative model is constructed out of the causal states by endowing the causal-state process with transitions:

$$T_{\sigma \sigma^{\prime }}^{\left(x\right)}=\text{Pr}(S_{t+1}^{+}=\sigma^{\prime },X_{t}=x|S_{t}^{+}=\sigma )\;,$$

that give the probability of generating the next symbol *x* and ending in the next state σ′, if starting in state σ (Residing in a state and generating a symbol do not occur simultaneously. Since symbols are generated during transitions there is, in effect, a half time-step difference in the indexes of the random variables *X*_*t*_ and $S_{t}^{+}$. We suppress notating this.) To summarize, a process's *forward-time* is the tuple {A, S^+^, {*T*^(*x*)^:*x* ∈ A}}.

For a discrete-time, discrete-alphabet process, the ϵ-machine is its minimal unifilar hidden Markov model (HMM) (Crutchfield and Young, 1989; Shalizi and Crutchfield, 2001) (For general background on HMMs see Paz, 1971; Rabiner and Juang, 1986; Rabiner, 1989). Note that the causal state set can be finite, countable, or uncountable; the latter two cases can occur even for processes generated by finite-state HMMs. *Minimality* can be defined by either the smallest number of states or the smallest entropy $H\left[S_{0}^{+}\right]$ over states (Shalizi and Crutchfield, 2001). *Unifilarity* is a constraint on the transition matrices *T*^(*x*)^ such that the next state σ′ is determined by knowing the current state σ and the next symbol *x*. That is, if the transition exists, then $\text{Pr}\left(S_{t+1}^{+}|X_{t}=x,S_{t}^{+}=\sigma \right)$ has support on a single causal state.

## 3. Infinitesimal time resolution

One often treats a continuous-time renewal process, such as a spike train from a noisy integrate-and-fire neuron, in a discrete-time setting (Rieke et al., 1999). With results of Marzen and Crutchfield (2015) in hand, we can investigate how artificial time binning affects estimates of a model neuron's spike train's randomness, predictability, and information storage in the limit of infinitesimal time resolution. This is exactly the limit in which analytic formulae for information measures are most useful, since increasing the time resolution artificially increases the apparent range of temporal correlations as shown in **Figure 3**.

Time-binned neural spike trains of noisy integrate-and-fire neurons have been studied for quite some time (Mackay and McCulloch, 1952) and, despite that history, this is still an active endeavor (Rieke et al., 1999; Cessac and Cofre, 2013). Our emphasis and approach differ, though. We do not estimate statistics or reconstruct models from simulated spike train data using nonparametric inference algorithms—e.g., as done in Haslinger et al. (2010). Rather, we ask how ϵ-machine extracted from a spike train process and information measures calculated from them vary as a function of time coarse-graining. Our analytic approach highlights an important lesson about such studies in general: A process' ϵ-machine and information anatomy are sensitive to time resolution. A secondary and compensating lesson is that the manner in which the ϵ-machine and information anatomy scale with time resolution conveys much about the process' structure.

Suppose we are given a neural spike train with interspike intervals independently drawn from the same interspike interval (ISI) distribution ϕ(*t*) with mean ISI 1/μ. To convert the continuous-time point process into a sequence of binary spike-quiescence symbols, we track the number of spikes emitted in successive time bins of size Δ*t*. Our goal, however, is to understand how the choice of Δ*t* affects reported estimates for *C*_μ_, *h*_μ_, **E**, *b*_μ_, and σ_μ_. The way in which each of these vary with Δ*t* reveals information about the intrinsic time scales on which a process behaves; cf., the descriptions of entropy rates in Costa et al. (2002, 2005) and Gaspard and Wang (1993). We concern ourselves with the infinitesimal Δ*t* limit, even though the behavior of these information atoms is potentially most interesting when Δ*t* is on the order of the process' intrinsic time scales.

In the infinitesimal time-resolution limit, when Δ*t* is smaller than any intrinsic timescale, the neural spike train is a renewal process with *interevent count distribution*:

(3) F(n)≈ϕ(nΔt) Δt

and *survival function*:

(4) $$w(n)\approx \int_{n\Delta t}^{\infty }\phi (t)dt\;.$$

The interevent distribution *F*(*n*) is the probability distribution that the silence separating successive events (bins with spikes) is *n* counts long. While the survival function *w*(*n*) is the probability that the silence separating successive events is at least *n* counts long. The ϵ-machine transition probabilities therefore change with Δ*t*. The mean interevent count 〈*T*〉 + 1 is *not* the mean interspike interval 1/μ since one must convert between counts and spikes^1^:

(5) $$\langle T\rangle +1=\frac{1}{\mu \Delta t}\;.$$

In this limit, the ϵ-machine of spike-train renewal processes can take one of the topologies described in Marzen and Crutchfield (2015).

**Figure 2:**
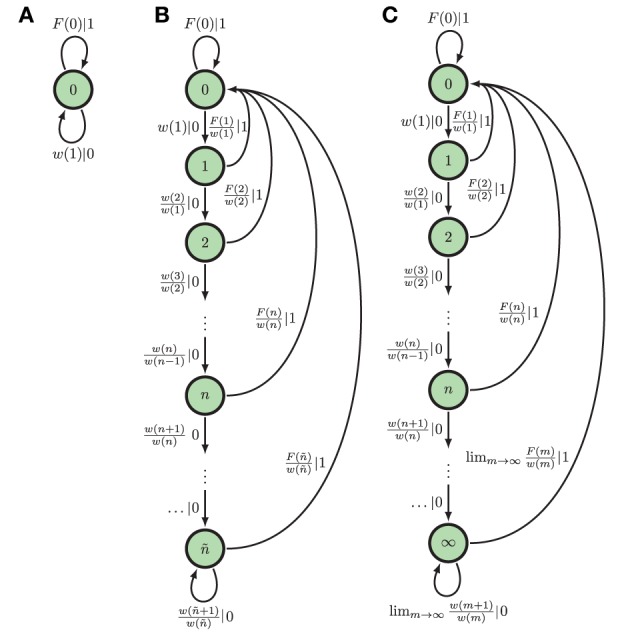
ϵ**-Machines of processes generated by Poisson neurons and by integrate-and-fire neurons (left to right): (A)** The ϵ-machine for a Poisson process. **(B)** The ϵ-machine for an eventually Poisson process; i.e., a Poisson neuron with a refractory period of length ñΔ*t*. **(C)** The ϵ-machine for a generic renewal process—the not eventually Δ-Poisson process of Marzen and Crutchfield (2015); i.e., the process generated by noise-driven integrate-and-fire neurons. Edge labels *p*|*x* denote emitting symbol *x* (“1” is “spike”) with probability *p*. (Reprinted with permission from Marzen and Crutchfield, 2015.)

Here, we focus only on two of these ϵ-machine topologies. The first topology corresponds to that of an eventually Poisson process, in which the ISI distribution takes the form ϕ(*t*) = ϕ(*T*)*e*^−λ(*t*−*T*)^ for some finite *T* and λ > 0. A Poisson neuron with firing rate λ and refractory period of time *T*, for instance, eventually (*t* > *T*) generates a Poisson process. Hence, we refer to them as *eventually Poisson processes*; see Figure 2B. A Poisson process is a special type of eventually Poisson process with *T* = 0; see Figure 2A. However, the generic renewal process has topology shown in Figure 2C. Technically, only non-eventually-Δ Poisson processes have this ϵ-machine topology, but for our purposes, this is the ϵ-machine topology for any renewal process *not* generated by a Poisson neuron; see Marzen and Crutchfield (2015).

At present, inference algorithms can only infer finite ϵ-machines. So, such algorithms applied to renewal processes will yield an eventually Poisson topology. (Compare Figure 2C to the inferred approximate of an integrate-and-fire neuron in Figure 2 in Haslinger et al., 2010.) The generic renewal process has an infinite ϵ-machines, though, for which the inferred ϵ-machines are only approximations.

We calculated **E** and *C*_μ_ using the expressions given in Marzen and Crutchfield (2015). Substituting in Equations (3), (4), and (5), we find that the excess entropy **E** tends to:

(6) $$\begin{array}{l} \underset{\Delta t\to 0}{\text{lim}}E(\Delta t)=\int_{0}^{\infty }\mu t\phi (t){\text{log}}_{2}(\mu \phi (t))dt\\ \;-\;2\int_{0}^{\infty }\mu \Phi (t){\text{log}}_{2}(\mu \Phi (t))dt\;, \end{array}$$

where $\Phi (t)=\int_{t}^{\infty }\phi (t^{\prime })dt^{\prime }$ is the probability that an ISI is longer than *t*. It is easy to see that **E**(Δ*t*) limits to a positive and (usually) finite value as the time resolution vanishes, with some exceptions described below. Similarly, using the expression in Marzen and Crutchfield (2015)'s Appendix II, one can show that the finite-time excess entropy^2^ **E**(*T*) takes the form:

(7) $$\begin{array}{l} \underset{\Delta t\to 0}{\text{lim}}E(T)=\left(\int_{0}^{T}\mu \Phi (t)dt\right){\text{log}}_{2}\frac{1}{\mu }\\ \;-\;2\int_{0}^{T}\mu \Phi (t){\text{log}}_{2}\Phi (t)dt\\ \;-\;\mu \int_{T}^{\infty }\Phi (t)dt{\text{log}}_{2}\left(\mu \int_{T}^{\infty }\Phi (t)dt\right)\\ \;+\int_{0}^{T}\mu tF(t){\text{log}}_{2}F(t)dt\\ \;+T\int_{T}^{\infty }\mu F(t){\text{log}}_{2}F(t)dt \end{array}$$

As *T* → ∞, **E**(*T*) → **E**. Note that these formulae apply only when mean firing rate μ is nonzero.

Even if **E** limits to a finite value, the statistical complexity typically diverges due to its dependence on time discretization Δ*t*. Suppose that we observe an eventually Poisson process, such that ϕ(*t*) = ϕ(*T*)*e*^−λ(*t*−*T*)^ for *t* > *T*. Then, from formulae in Marzen and Crutchfield (2015), statistical complexity in the infinitesimal time-resolution limit becomes:

(8) $$\begin{array}{l} C_{\mu }(\Delta t)\sim \left(\mu \int_{0}^{T}\Phi (t)dt\right){\text{log}}_{2}\frac{1}{\Delta t}\\ \;-\int_{0}^{T}\left(\mu \Phi (t)\right){\text{log}}_{2}(\mu \Phi (t))dt\\ \;-\left(\mu \int_{T}^{\infty }\Phi (t)dt\right){\text{log}}_{2}\left(\mu \int_{T}^{\infty }\Phi (t)dt\right), \end{array}$$

ignoring terms of *O*(Δ*t*) or higher. The first term diverges, and its rate of divergence is the probability of observing a time since last spike less than *T*. This measures the spike train's deviation from being Δ-Poisson and so reveals the effective dimension of the underlying causal state space. *C*_μ_'s remaining nondivergent component is equally interesting. In fact, it is the differential entropy of the time since last spike distribution.

An immediate consequence of the analysis is that this generic infinitesimal renewal process is highly *cryptic* (Crutchfield et al., 2009). It hides an arbitrarily large amount of its internal state information: *C*_μ_ diverges as Δ*t* → 0 but **E** (usually) asymptotes to a finite value. We have very structured processes that have disproportionately little in the future to predict. Periodic processes constitute an important exception to this general rule of thumb for continuous-time processes. A neuron that fires every *T* seconds without jitter has **E** = *C*_μ_, and both **E** and *C*_μ_ diverge logarithmically with 1/Δ*t*.

It is straightforward to show that any information measure contained within the present—*H*[*X*_0_], *h*_μ_, *b*_μ_, *r*_μ_, and *q*_μ_ (recall Figure 1)—all vanish as Δ*t* tends to 0. Therefore, ${\text{lim}}_{\Delta t\to 0}\sigma_{\mu }={\text{lim}}_{\Delta t\to 0}$ **E** and the entropy rate becomes:

(9) $$h_{\mu }\sim -\mu \left({\text{log}}_{2}(\Delta t)+\int_{0}^{\infty }\phi (t){\text{log}}_{2}\phi (t)dt\right)\Delta t\;.$$

With Δ*t* → 0, *h*_μ_ nominally tends to 0: As we shorten the observation time scale, spike events become increasingly rare. There are at least two known ways to address *h*_μ_ apparently not being very revealing when so defined. On the one hand, rather than focusing on the uncertainty per symbol, as *h*_μ_ does, we opt to look at the uncertainty per unit time: *h*_μ_/Δ*t*. This is the so-called Δ*t-entropy rate* (Gaspard and Wang, 1993) and it diverges as −μ log Δ*t*. Such divergences are to be expected: The large literature on dimension theory characterizes a continuous set's randomness by its divergence scaling rates (Farmer et al., 1983; Mayer-Kress, 1986). Here, we are characterizing sets of similar cardinality—infinite sequences. On the other hand, paralleling sequence block-entropy definition of entropy rate (*h*_μ_ =_ℓ → ∞_*H*[*X*_0:ℓ_]/ℓ) (Crutchfield and Feldman, 2003), continuous-time entropy rates are often approached within a continuous-time framework using:

$$h_{\mu }=\underset{T\to \infty }{\text{lim}}H(T)/T\;,$$

where *H*(*T*) is path entropy, the continuous-time analog of the block entropy *H*(ℓ) (Girardin, 2005). In these analyses, any log Δ*t* terms are regularized away using Shannon's differential entropy (Cover and Thomas, 2006), leaving the nondivergent component $-\mu \int_{0}^{\infty }\phi \left(t\right)\log\phi \left(t\right)dt$. Using the Δ*t*-entropy rate but keeping both the divergent and nondivergent components, as in Equations (8) and (9), is an approach that respects both viewpoints and gives a detailed picture of time-resolution scaling.

A major challenge in analyzing spike trains concerns locating the timescales on which information relevant to the stimulus is carried. Or, more precisely, we are often interested in estimating what percentage of the raw entropy of a neural spike train is used to communicate information about a stimulus; cf. the framing in Strong et al. (1998). For such analyses, the entropy rate is often taken to be *H*(Δ*t, T*)/*T*, where *T* is the total path time and *H*(Δ*t, T*) is the entropy of neural spike trains over time *T* resolved at time bin size Δ*t*. In terms of previously derived quantities and paralleling the well known block-entropy linear asymptote *H*(ℓ) = **E** + *h*_μ_ℓ (Crutchfield and Feldman, 2003), this is:

$$\frac{H(\Delta t,T)}{T}=\frac{h_{\mu }(\Delta t)}{\Delta t}+\frac{E(T,\Delta t)}{T}\;.$$

From the scaling analyses above, the extensive component of *H*(Δ*t, T*)/*T* diverges logarithmically in the small Δ*t* limit due to the logarithmic divergence (Equation 9) in *h*_μ_(Δ*t*)/Δ*t*. If we are interested in accurately estimating the entropy rate, then the above is one finite-time *T* estimate of it. However, there are other estimators, including:

$$\frac{H(\Delta t,T)-H(\Delta t,T-\Delta t)}{\Delta t}\approx \frac{h_{\mu }(\Delta t)}{\Delta t}+\frac{\partial E(T,\Delta t)}{\partial T}\;.$$

This estimator converges more quickly to the true entropy rate *h*_μ_(Δ*t*)/Δ*t* than does *H*(Δ*t, T*)/*T*.

No such log Δ*t* divergences occur with *b*_μ_. Straightforward calculation, not shown here, reveals that:

(10) $$\begin{array}{l} \underset{\Delta t\to 0}{\text{lim}}\frac{b_{\mu }}{\Delta t}=-\mu (\int_{0}^{\infty }\phi (t)\int_{0}^{\infty }\phi (t^{\prime }){\text{log}}_{2}\phi (t+t^{\prime })dt^{\prime }dt\\ \;+\;\frac{1}{\text{log}2}-\int_{0}^{\infty }\phi (t){\text{log}}_{2}\phi (t)dt)\;. \end{array}$$

Since ${\text{lim}}_{\Delta t\to 0}b_{\mu }(\Delta t)/\Delta t\;<\;\infty$ and ${\text{lim}}_{\Delta t\to 0}h_{\mu }(\Delta t)/\Delta t\;$ diverges, the ephemeral information rate *r*_μ_(Δ*t*)/Δ*t* also diverges as Δ*t* → 0. The bulk of the information generated by such renewal processes is dissipated and, having no impact on future behavior, is not useful for prediction.

Were we allowed to observe relatively microscopic membrane voltage fluctuations rather than being restricted to the relatively macroscopic spike sequence, the Δ*t*-scaling analysis would be entirely different. Following Marzen and Crutchfield (2014) or natural extensions thereof, the statistical complexity diverges as −log ϵ, where ϵ is the resolution level for the membrane voltage, the excess entropy diverges as log1/Δ*t*, the time-normalized entropy rate diverges as $\log\sqrt{2\pi eD\Delta t}∕\Delta t$, and the time-normalized bound information diverges as 1/2Δ*t*. In other words, observing membrane voltage rather than spikes makes the process far more predictable. The relatively more macroscopic modeling at the level of spikes throws away much detail of the underlying biochemical dynamics.

To illustrate the previous points, we turn to numerics and a particular neural model. Consider an (unleaky) integrate-and-fire neuron driven by white noise whose membrane voltage (after suitable change of parameters) evolves according to:

(11) $$\frac{dV}{dt}=b+\sqrt{D}\eta (t),$$

where η(*t*) is white noise such that 〈η(*t*)〉 = 0 and 〈η(*t*)η(*t*′)〉 = δ(*t* − *t*′). When *V* = 1, the neuron spikes and the voltage is reset to *V* = 0; it stays at *V* = 0 for a time τ, which enforces a hard refractory period. Since the membrane voltage resets to a predetermined value, the interspike intervals produced by this model are independently drawn from the same interspike interval distribution:

(12) $$\phi (t)=\{\begin{array}{ll} 0 & t<\tau \\ \sqrt{\frac{\lambda }{2\pi {\left(t-\tau \right)}^{3}}}e^{-\lambda {\left(\mu (t-\tau )-1\right)}^{2}/2(t-\tau )} & t\geq \tau \end{array}\;.$$

Here, 1/μ = 1/*b* is the mean interspike interval and λ = 1/*D* is a shape parameter that controls ISI variance. This neural model is not as realistic as that of a linear leaky integrate-and-fire neural model (Gerstner and Kistler, 2002), but is complex enough to illustrate the points made earlier about the scaling of information measures and time resolution.

For illustration purposes, we assume that the time-binned neural spike train is well approximated by a renewal process, even when Δ*t* is as large as one millisecond. This assumption will generally not hold, as past interevent counts could provide more detailed historical information that more precisely places the last spike within its time bin. Even so, the reported information measure estimates are still useful. The estimated *h*_μ_ is an upper bound on the true entropy rate; the reported **E** is a lower bound on the true excess entropy using the Data Processing Inequality (Cover and Thomas, 2006); and the reported *C*_μ_ will usually be a lower bound on the true process' statistical complexity.

**Figure 3:**
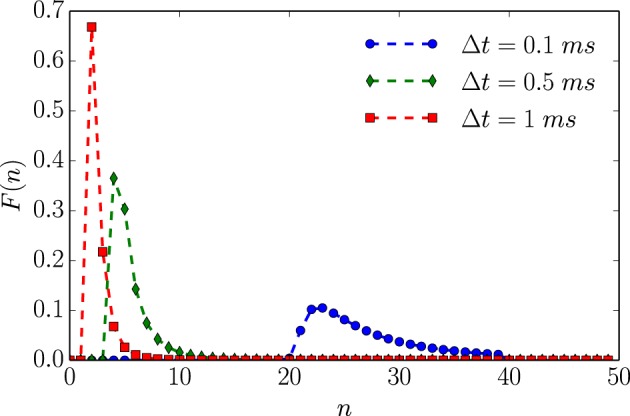
**An unleaky integrate-and-fire neuron driven by white noise has varying interevent count distributions *F*(*n*) that depend on time bin size Δ*t***. Based on the ISI distribution ϕ(*t*) given in Equation (12) with τ = 2 ms, 1/μ = 1 ms, and λ = 1 ms. Data points represent exact values of *F*(*n*) calculated for integer values of *N*. Dashed lines are interpolations based on straight line segments connecting nearest neighbor points.

**Figure 4:**
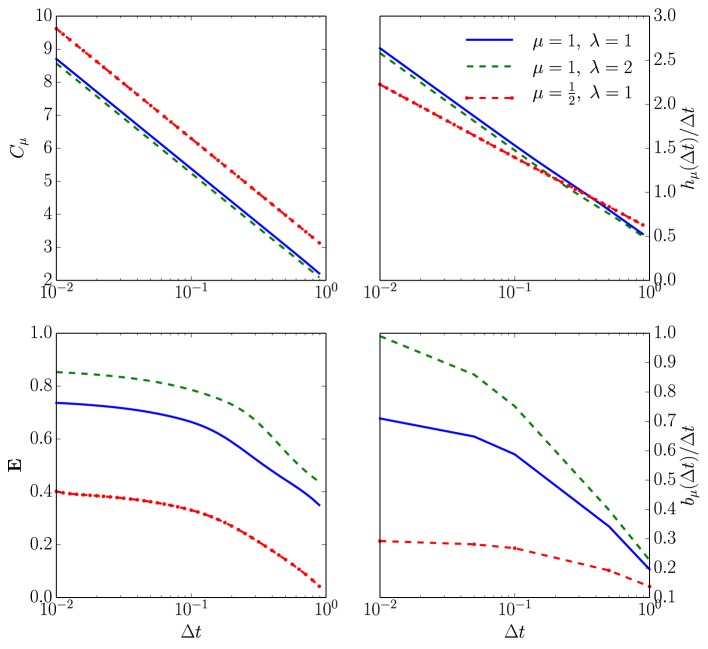
**How spike-train information measures (or rates) depend on time discretization Δ*t* for an unleaky integrate-and-fire neuron driven by white noise**. **Top left:** Statistical complexity *C*_μ_ as a function of both the ISI distribution shape parameters and the time bin size Δ*t*. The horizontal axis is Δ*t* in milliseconds on a log-scale and the vertical axis is *C*_μ_ in bits on a linear scale for three different ISI distributions following Equation (12) with τ = 2 ms. **Top right:** Entropy rate *h*_μ_ also as a function of both shape parameters and Δ*t*. Axes labeled as in the previous panel and the same three ISI distributions are used. **Bottom left:** Excess entropy *E* as a function of both the shape parameters and Δ*t*. For the blue line ${\text{lim}}_{\Delta t\to 0}\text{E}\left(\Delta t\right)=0.75$ bits; purple line, ${\text{lim}}_{\Delta t\to 0}\text{E}\left(\Delta t\right)=0.86$ bits; and yellow line, ${\text{lim}}_{\Delta t\to 0}\text{E}\left(\Delta t\right)=0.41$ bits. All computed from Equation (6). **Bottom right:** Bound information rate *b*_μ_(Δ*t*)/Δ*t* parameterized as in the previous panels. For the blue line ${\text{lim}}_{\Delta t\to 0}b_{\mu }\left(\Delta t\right)∕\Delta t=0.73$ bits per second; purple line, ${\text{lim}}_{\Delta t\to 0}b_{\mu }\left(\Delta t\right)∕\Delta t=1.04$ bits per second; and yellow line, ${\text{lim}}_{\Delta t\to 0}b_{\mu }\left(\Delta t\right)∕\Delta t=0.30$ bits per second. All computed from Equation (10).

Employing the renewal process assumption, numerical analysis corroborates the infinitesimal analysis above. Figure 3 plots *F*(*n*)—the proxy for the full, continuous-time, ISI distribution—for a given set of neuronal parameter values as a function of time resolution. Figure 4 then shows that *h*_μ_ and *C*_μ_ exhibit logarithmic scaling at millisecond time discretizations, but that **E** does not converge to its continuous-time value until we reach time discretizations on the order of hundreds of microseconds. Even when Δ*t* = 100 μ*s*, *b*_μ_(Δ*t*)/Δ*t* still has not converged to its continuous-time values.

The statistical complexity *C*_μ_ increases without bound, as Δ*t* → 0; see the top left panel of Figure 4. As suggested in the infinitesimal renewal analysis, *h*_μ_ vanishes, whereas *h*_μ_/Δ*t* diverges at a rate of μlog_2_1/Δ*t*, as shown in the top right plots of Figure 4. As anticipated, **E** tends to a finite, ISI distribution-dependent value when Δ*t* tends to 0, as shown in the bottom left panel in Figure 4. Finally, the lower right panel plots *b*_μ_(Δ*t*)/Δ*t*.

One conclusion from this simple numerical analysis is that one should consider going to submillisecond time resolutions to obtain accurate estimates of ${\text{lim}}_{\Delta t\to 0}E(\Delta t)$ and ${\text{lim}}_{\Delta t\to 0}b_{\mu }(\Delta t)/\Delta t\;$, even though the calculated informational values are a few bits or even less than one bit per second in magnitude.

## 4. Alternating renewal processes

The form of the Δ*t*-scalings discussed in Section 3 occur much more generally than indicated there. Often, our aim is to calculate the nondivergent component of these information measures as Δ*t* → 0, but the rates of these scalings are process-dependent. Therefore, these divergences can be viewed as a feature rather than a bug; they contain additional information about the process' structure (Gaspard and Wang, 1993).

To illustrate this point, we now investigate Δ*t*-scalings for information measures of alternating renewal processes (ARPs), which are structurally more complex than the standard renewal processes considered above. For instance, these calculations suggest that rates of divergence of the τ-entropy rate smaller than the firing rate, such as those seen in Nemenman et al. (2008), are indicative of strong ISI correlations. Calculational details are sequestered in Appendix A.

**Figure 5:**
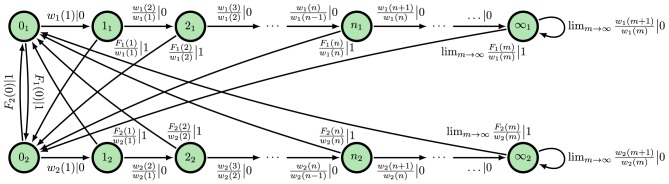
**ϵ-Machine for an alternating renewal process in which neither interevent count distribution is Δ-Poisson and they are not equal almost everywhere**. State label *n*_*m*_ denotes *n* counts since the last event and present modality *m*.

In an ARP, an ISI is drawn from one distribution ϕ^(1)^(*t*), then another distribution ϕ^(2)^(*t*), then the first ϕ^(1)^(*t*) again, and so on. We refer to the new piece of additional information—the ISI distribution currently being drawn from—as the *modality*. Under weak technical conditions, the causal states are the modality and time since last spike. The corresponding, generic ϵ-machine is shown in Figure 5. We define the modality-dependent survival functions as $\Phi_{i}\left(t\right)=\int_{t}^{\infty }\phi^{\left(i\right)}\left(t^{\prime }\right)dt^{\prime }$, the modality-dependent mean firing rates as:

(13) $$\mu^{\left(i\right)}=1/\int_{0}^{\infty }\phi^{\left(i\right)}(t)dt,$$

the modality-dependent differential entropy rates:

$$h_{\mu }^{\left(i\right)}=-\mu^{\left(i\right)}\int_{0}^{\infty }\phi^{\left(i\right)}{\text{log}}_{2}\phi^{\left(i\right)}(t)dt\;,$$

the modality-dependent continuous-time statistical complexity:

$$C_{\mu }^{\left(i\right)}=-\int_{0}^{\infty }\mu^{\left(i\right)}\Phi^{\left(i\right)}(t){\text{log}}_{2}\left(\mu^{\left(i\right)}\Phi^{\left(i\right)}(t)\right)dt\;,$$

and the modality-dependent excess entropy:

(14) $$\begin{array}{l} E^{\left(i\right)}=\int_{0}^{\infty }\mu^{\left(i\right)}t\phi^{\left(i\right)}(t){\text{log}}_{2}\left(\mu^{\left(i\right)}\phi^{\left(i\right)}(t)\right)dt\\ \;-\;2\int_{0}^{\infty }\mu^{\left(i\right)}\Phi^{\left(i\right)}(t){\text{log}}_{2}\left(\mu^{\left(i\right)}\Phi^{\left(i\right)}(t)\right)dt\;. \end{array}$$

It is straightforward to show, as done in Appendix A, that the time-normalized entropy rate still scales with log_2_1/Δ*t*:

(15) $$\frac{h_{\mu }(\Delta t)}{\Delta t}\text{​}\sim \text{​}\frac{1}{2}\mu {\text{log}}_{2}\left(\frac{1}{\Delta t}\right)\text{​}+\text{​}\frac{\mu^{\left(2\right)}h_{\mu }^{\left(1\right)}\text{​}+\;\mu^{\left(1\right)}h_{\mu }^{\left(2\right)}}{\mu^{\left(1\right)}+\mu^{\left(2\right)}},$$

where $\mu =2\frac{\mu^{\left(1\right)}\mu^{\left(2\right)}}{\mu^{\left(1\right)}+\mu^{\left(2\right)}}$. As expected, the statistical complexity still diverges:

(16) $$\begin{array}{l} C_{\mu }(\Delta t)\sim 2{\text{log}}_{2}\left(\frac{1}{\Delta t}\right)+\frac{\mu^{\left(2\right)}C_{\mu }^{\left(1\right)}+\mu^{\left(1\right)}C_{\mu }^{\left(2\right)}}{\mu^{\left(1\right)}+\mu^{\left(2\right)}}\\ \;+\;H_{b}\left(\frac{\mu_{1}}{\mu_{1}+\mu_{2}}\right)\;, \end{array}$$

where *H*_*b*_(*p*) = −*p* log_2_ *p* − (1 − *p*) log_2_ (1 − *p*) is the entropy in bits of a Bernoulli random variable with bias *p*. Finally, the excess entropy still limits to a positive constant:

(17) $$\underset{\Delta t\to 0}{\text{lim}}E(\Delta t)=H_{b}\left(\frac{\mu_{1}}{\mu_{1}+\mu_{2}}\right)+\frac{\mu^{\left(2\right)}E^{\left(1\right)}+\mu^{\left(1\right)}E^{\left(2\right)}}{\mu^{\left(1\right)}+\mu^{\left(2\right)}}\;.$$

The additional terms *H*_*b*_(·) come from the information stored in the time course of modalities.

As a point of comparison, we ask what these information measures would be for the original (noncomposite) renewal process with the same ISI distribution as the ARP. As described in Appendix B, the former entropy rate is always less than the true *h*_μ_; its statistical complexity is always less than the true *C*_μ_; and its excess entropy is always smaller than the true ***E***. In particular, the ARP's *h*_μ_ divergence rate is always less than or equal to the mean firing rate μ. Interestingly, this coincides with what was found empirically in the time series of a single neuron; see Figure 5C in Nemenman et al. (2008).

The ARPs here are a first example of how one can calculate information measures of the much broader and more structurally complex class of processes generated by unifilar hidden semi-Markov models, a subclass of hidden semi-Markov models (Tokdar et al., 2010).

## 5. Information universality

Another aim of ours is to interpret the information measures. In particular, we wished to relate infinitesimal time-resolution excess entropies, statistical complexities, entropy rates, and bound information rates to more familiar characterizations of neural spike trains—firing rates μ and ISI coefficient of variations *C*_*V*_. To address this, we now analyze a suite of familiar single-neuron models. We introduce the models first, describe the parameters behind our numerical estimates, and then compare the information measures.

Many single-neuron models, when driven by temporally uncorrelated and stationary input, produce neural spike trains that are renewal processes. We just analyzed one model class, the noisy integrate-and-fire (NIF) neurons in Section 3, focusing on time-resolution dependence. Other common neural models include the linear leaky integrate-and-fire (LIF) neuron, whose dimensionless membrane voltage, after a suitable change of parameters, fluctuates as:

(18) $$\frac{dV}{dt}=b-V+a\eta (t)\;,$$

and when *V* = 1, a spike is emitted and *V* is instantaneously reset to 0. We computed ISI survival functions from empirical histograms of 10^5^ ISIs; we varied *b* ∈ [1.5, 5.75] in steps of 0.25 and *a* ∈ [0.1, 3.0] in steps of 0.1 to *a* = 1.0 and in steps of 0.25 thereafter.

The quadratic integrate-and-fire (QIF) neuron has membrane voltage fluctuations that, after a suitable change of variables, are described by:

(19) $$\frac{dV}{dt}=b+V^{2}+a\eta (t)\;,$$

and when *V* = 100, a spike is emitted and *V* is instantaneously reset to −100. We computed ISI survival functions from empirical histograms of trajectories with 10^5^ ISIs; we varied *b* ∈ [0.25, 4.75] in steps of 0.25 and *a* ∈ [0.25, 2.75] in steps of 0.25. The QIF neuron has a very different dynamical behavior from the LIF neuron, exhibiting a Hopf bifurcation at *b* = 0. Simulation details are given in Appendix B.

Finally, ISI distributions are often fit to gamma distributions, and so we also calculated the information measures of spike trains with gamma-distributed ISIs (GISI).

Each neural model—NIF, LIF, QIF, and GISI—has its own set of parameters that governs its ISI distribution shape. Taken at face value, this would make it difficult to compare information measures across models. Fortunately, for each of these neural models, the firing rate μ and coefficient of variation *C*_*V*_ uniquely determine the underlying model parameters (Vilela and Lindner, 2009). As Appendix B shows, the quantities $\lim_{\Delta t\to 0}E(\Delta t)$, $\lim_{\Delta t\to 0}C_{\mu }+\log_{2}(\mu \Delta t)$, $\lim_{\Delta t\to 0}h_{\mu }(\Delta t)/\mu \Delta t+\log_{2}(\mu \Delta t)$, and $\lim_{\Delta t\to 0}b_{\mu }(\Delta t)/\mu \Delta t$ depend only on the ISI coefficient of variation *C*_*V*_ and not the mean firing rate μ.

We estimated information measures from the simulated spike train data using plug-in estimators based on the formulae in Section 3. Enough data was generated that even naive plug-in estimators were adequate *except* for estimating *b*_μ_ when *C*_*V*_ was larger than 1. See Appendix B for estimation details. That said, binned estimators are likely inferior to binless entropy estimators (Victor, 2002), and naive estimators tend to have large biases. This will be an interesting direction for future research, since a detailed analysis goes beyond the present scope.

**Figure 6:**
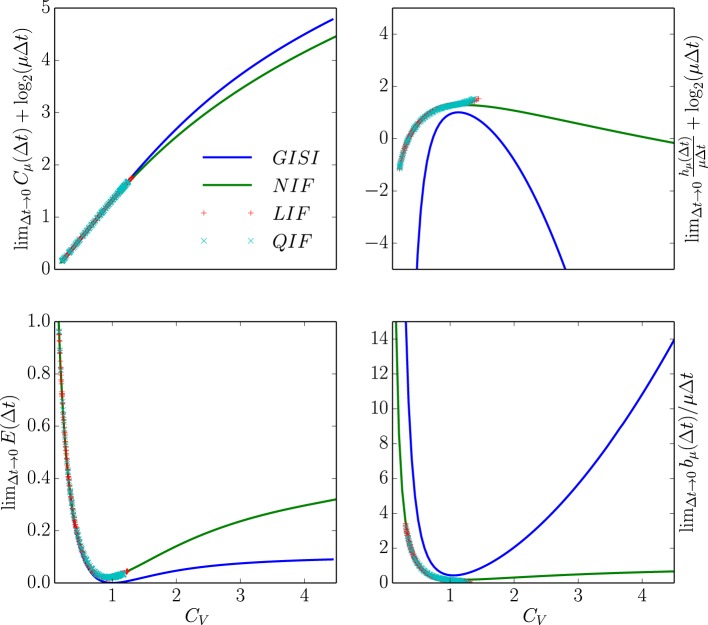
**Information universality across distinct neuron dynamics**. We find that several information measures depend only on the ISI coefficient of variation *C*_*V*_ and not the ISI mean firing rate μ for the following neural spike train models: (i) neurons with Gamma distributed ISIs (GISI, blue), (ii) noisy integrate-and-fire neurons governed by Equation (11) (NIF, green), (iii) noisy linear leaky integrate-and-fire neurons governed by Equation (18) (LIF, dotted red), and (iv) noisy quadratic integrate-and-fire neurons governed by Equation (19) (QIF, dotted blue). **Top left:** ${\text{lim}}_{\Delta t\to 0}C_{\mu }\left(\Delta t\right)+\log_{2}\left(\mu \Delta t\right)$. **Top right:** ${\text{lim}}_{\Delta t\to 0}h_{\mu }\left(\Delta t\right)∕\mu \Delta t+\log_{2}\left(\mu \Delta t\right)$. **Bottom left:** ${\text{lim}}_{\Delta t\to 0}\text{E}\left(\Delta t\right)$. **Bottom right:** $\underset{\Delta t\to 0}{\lim}b_{\mu }\left(\Delta t\right)∕\mu \Delta t)$. In the latter, ISI distributions with smaller *C*_*V*_ were excluded due to the difficulty of accurately estimating $\int_{0}^{\infty }\int_{0}^{\infty }\phi \left(t\right)\phi \left(t^{\prime }\right)\log_{2}\phi \left(t+t^{\prime }\right)dtdt^{\prime }$ from simulated spike trains. See text for discussion.

Figure 6 compares the statistical complexity, excess entropy, entropy rate, and bound information rate for all four neuron types as a function of their *C*_*V*_. Surprisingly, the NIF, LIF, and QIF neuron's information measures have essentially identical dependence on *C*_*V*_. That is, the differences in mechanism do not strongly affect these informational properties of the spike trains they generate. Naturally, this leads one to ask if the informational indifference to mechanism generalizes to other spike train model classes and stimulus-response settings.

Figure 6's top left panel shows that the continuous-time statistical complexity grows monotonically with increasing *C*_*V*_. In particular, the statistical complexity increases logarithmically with ISI mean and approximately linearly with the ISI coefficient of variation *C*_*V*_. That is, the number of bits that must be stored to predict these processes increases in response to additional process stochasticity and longer temporal correlations. In fact, it is straightforward to show that the statistical complexity is minimized and excess entropy maximized at fixed μ when the neural spike train is periodic. This is unsurprising since, in the space of processes, periodic processes are least cryptic (*C*_μ_ − **E** = 0) and so knowledge of oscillation phase is enough to completely predict the future. (See Appendix B.)

The bottom left panel in Figure 6 shows that increasing *C*_*V*_ tends to decrease the excess entropy **E**—the number of bits that one can predict about the future. **E** diverges for small *C*_*V*_, dips at the *C*_*V*_ where the ISI distribution is closest to exponential, and limits to a small number of bits at large *C*_*V*_. At small *C*_*V*_, the neural spike train is close to noise-free periodic behavior. When analyzed at small but nonzero Δ*t*, **E** encounters an “ultraviolet divergence” (Tchernookov and Nemenman, 2013). Thus, **E** diverges as *C*_*V*_ → 0, and a simple argument in Appendix B suggests that the rate of divergence is log_2_(1/*C*_*V*_). At an intermediate *C*_*V*_ ~ 1, the ISI distribution is as close as possible to that of a memoryless Poisson process and so **E** is close to vanishing. At larger *C*_*V*_, the neural spike train is noise-driven. Surprisingly, completely noise-driven processes still have a fraction of a bit of predictability: knowing the time since last spike allows for some power in predicting the time to next spike.

The top right panel shows that an appropriately rescaled differential entropy rate varies differently for neural spike trains from noisy integrate-and-fire neurons and neural spike trains with gamma-distributed ISIs. As expected, the entropy rate is maximized at *C*_*V*_ near 1, consistent with the Poisson process being the maximum entropy distribution for fixed mean ISI. Gamma-distributed ISIs are far less random than ISIs from noisy integrate-and-fire neurons, holding μ and *C*_*V*_ constant.

Finally, the continuous-time bound information (*b*_μ_) rate varies in a similar way to **E** with *C*_*V*_. (Note that since the plotted quantity is ${\text{lim}}_{\Delta t\to 0}b_{\mu }$, one could interpret the normalization by 1/μ as a statement about how the mean firing rate μ sets the natural timescale.) At low *C*_*V*_, the *b*_μ_ rate diverges as $1∕C_{V}^{2}$, as described in Appendix B. Interestingly, this limit is singular, similar to the results in Marzen and Crutchfield (2014): at *C*_*V*_ = 0, the spike train is noise-free periodic and so the *b*_μ_ rate is 0. For *C*_*V*_ ≈ 1, it dips for the same reason that **E** decreases. For larger *C*_*V*_, *b*_μ_'s behavior depends rather strongly on the ISI distribution shape. The longer-ranged gamma-distribution results in ever-increasing *b*_μ_ rate for larger *C*_*V*_, while the *b*_μ_ rate of neural spike trains produced by NIF neurons tends to a small positive constant at large *C*_*V*_. The variation of *b*_μ_ deviates from that of **E** qualitatively at larger *C*_*V*_ in that the GISI spike trains yield smaller total predictability **E** than that of NIF neurons, but arbitrarily higher predictability *rate*.

These calculations suggest a new kind of universality for neuronal information measures *within a particular generative model class*. All of these distinct integrate-and-fire neuron models generate ISI distributions from different families, yet their informational properties exhibit the same dependencies on Δ*t*, μ, and *C*_*V*_ in the limit of small Δ*t*. Neural spike trains with gamma-distributed ISIs did not show similar informational properties. And, we would not expect neural spike trains that are alternating renewal processes to show similar informational properties either. (See Section 4.) These coarse information quantities might therefore be effective model selection tools for real neural spike train data, though more groundwork must be explored to ascertain their utility.

## 6. Conclusions

We explored the scaling properties of a variety of information-theoretic quantities associated with two classes of spiking neural models: renewal processes and alternating renewal processes. We found that information generation (entropy rate) and stored information (statistical complexity) both diverge logarithmically with decreasing time resolution for both types of spiking models, whereas the predictable information (excess entropy) and active information accumulation (bound information rate) limit to a constant. Our results suggest that the excess entropy and regularized statistical complexity of different types of integrate-and-fire neurons are universal in the sense that they do not depend on mechanism details, indicating a surprising simplicity in complex neural spike trains. Our findings highlight the importance of analyzing the scaling behavior of information quantities, rather than assessing these only at a fixed temporal resolution.

By restricting ourselves to relatively simple spiking models we have been able to establish several key properties of their behavior. There are, of course, other important spiking models that cannot be expressed as renewal processes or alternating renewal processes, but we are encouraged by the robust scaling behavior of the entropy rate, statistical complexity, excess entropy, and bound information rate over the range of models we considered.

There was a certain emphasis here on the entropy rate and hidden Markov models of neural spike trains, both familiar tools in computational neuroscience. On this score, our contributions are straightforward. We determined how the entropy rate varies with the time discretization and identified the possibly infinite-state, unifilar HMMs required for optimal prediction of spike-train renewal processes. Entropy rate diverges logarithmically for stochastic processes (Gaspard and Wang, 1993), and this has been observed empirically for neural spike trains for time discretizations in the submillisecond regime (Nemenman et al., 2008). We argued that the *h*_μ_ divergence rate is an important characteristic. For renewal processes, it is the mean firing rate; for alternating renewal processes, the “reduced mass” of the mean firing rates. Our analysis of the latter, more structured processes showed that a divergence rate less than the mean firing rate—also seen experimentally (Nemenman et al., 2008)—indicates that there are strong correlations between ISIs. Generally, the nondivergent component of the time discretization-normalized entropy rate is the differential entropy rate; e.g., as given in Stevens and Zador (1996).

Empirically studying information measures as a function of time resolution can lead to a refined understanding of the time scales over which neuronal communication occurs. Regardless of the information measure chosen, the results and analysis here suggest that much can be learned by studying scaling behavior rather than focusing only on neural information as a single quantity estimated at a fixed temporal resolution. While we focused on the regime in which the time discretization was smaller than any intrinsic timescale of the process, future and more revealing analyses would study scaling behavior at even smaller time resolutions to directly determine intrinsic time scales (Crutchfield, 1994).

Going beyond information generation (entropy rate), we analyzed information measures—namely, statistical complexity and excess entropy—that have only recently been used to understand neural coding and communication. Their introduction is motivated by the hypothesis that neurons benefit from learning to predict their inputs (Palmer et al., 2013), which can consist of the neural spike trains of upstream neurons. The statistical complexity is the minimal amount of historical information required for exact prediction. To our knowledge, the statistical complexity has appeared only once previously in computational neuroscience (Haslinger et al., 2010). The excess entropy, a closely related companion, is the maximum amount of information that can be predicted about the future. When it diverges, then its divergence rate is quite revealing of the underlying process (Crutchfield, 1994; Bialek et al., 2001), but none of the model neural spike trains studied here had divergent excess entropy. Finally, the bound information rate has yet to be deployed in the context of neural coding, though related quantities have drawn attention elsewhere, such as in nonlinear dynamics (James et al., 2014), music (Abdallah and Plumbley, 2009), spin systems (Abdallah and Plumbley, 2012), and information-based reinforcement learning (Martius et al., 2013). Though its potential uses have yet to be exploited, it is an interesting quantity in that it captures the rate at which spontaneously generated information is actively stored by neurons. That is, it quantifies how neurons harness randomness.

Our contributions to this endeavor are more substantial than the preceding points. We provided exact formulae for the above quantities for renewal processes and alternating renewal processes. The new expressions can be developed further as lower bounds and empirical estimators for a process' statistical complexity, excess entropy, and bound information rate. This parallels how the renewal-process entropy-rate formula is a surprisingly accurate entropy-rate estimator (Gao et al., 2008). By deriving explicit expressions, we were able to analyze time-resolution scaling, showing that the statistical complexity diverges logarithmically for all but Poisson processes. So, just like the entropy rate, any calculations of the statistical complexity—e.g., as in Haslinger et al. (2010)—should be accompanied by the time discretization dependence. Notably, the excess entropy and the bound information rate have no such divergences.

To appreciate more directly what neural information processing behavior these information measures capture in the continuous-time limit, we studied them as functions of the ISI coefficient of variation. With an appropriate renormalization, simulations revealed surprising simplicity: a universal dependence on the coefficient of variation across several familiar neural models. The simplicity is worth investigating further since the dynamics and biophysical mechanisms implicit in the alternative noisy integrate-and-fire neural models are quite different. If other generative models of neural spike trains also show similar information universality, then these information measures might prove useful as model selection tools.

Finally, we close with a discussion of a practical issue related to the scaling analyses—one that is especially important given the increasingly sophisticated neuronal measurement technologies coming online at a rapid pace (Alivisatos et al., 2012). How small should Δ*t* be to obtain correct estimates of neuronal communication? First, as we emphasized, there is no single “correct” estimate for an information quantity, rather its resolution scaling is key. Second, results presented here and in a previous study by others (Nemenman et al., 2008) suggest that extracting information scaling rates and nondivergent components can require submillisecond time resolution. Third, and to highlight, the regime of infinitesimal time resolution is exactly the limit in which computational efforts without analytic foundation will fail or, at a minimum, be rather inefficient. As such, we hope that the results and methods developed here will be useful to these future endeavors and guide how new technologies facilitate scaling analysis.

### Conflict of interest statement

The authors declare that the research was conducted in the absence of any commercial or financial relationships that could be construed as a potential conflict of interest.

## Footnotes

^1^As the subscript context makes clear, the mean count μ is not related to that μ in *C*_μ_ and related quantities. In the latter it refers to the measure μ(**s**) over bi-infinite sequences **s** generated by a process.

^2^In the theoretical neuroscience literature, **E**(*T*) is sometime called the predictive information *I*_*pred*_(*T*) and is a useful indicator of process complexity when **E** diverges (Bialek et al., 2001).

## Appendix A

### Alternating renewal process information measures

A discrete-time alternating renewal process draws counts from *F*_1_(*n*), then *F*_2_(*n*), then *F*_1_(*n*), and so on. We now show that the modality and counts since last event are causal states when *F*_1_ ≠ *F*_2_ almost everywhere and when neither *F*_1_ nor *F*_2_ is eventually Δ-Poisson. We present only a proof sketch.

Two pasts *x*_:0_ and $x_{:0}^{\prime }$ belong to the same causal state when $\text{Pr}\left(X_{0:}|X_{:0}=x_{:0}\right)=\text{Pr}\left(X_{0:}|X_{:0}=x_{:0}^{\prime }\right)$. We can describe the future uniquely by a sequence of interevent counts N_*i*_, *i* ≥ 1, and the counts till next event $N_{0}^{\prime }$. Likewise, we could describe the past as a sequence of interevent counts N_*i*_, *i* < 0, and the counts since last event $N_{0}-N_{0}^{\prime }$. Let M_*i*_ be the modality at time step *i*. So, for instance, M_0_ is the present modality.

First, we claim that one can infer the present modality from a semi-infinite past almost surely. The probability that the present modality is 1 having observed the last 2*M* events is:

$$\begin{array}{l} \text{Pr}(M_{0}=1|N_{-2M:-1}=n_{-2M:-1})\\ \;=\prod_{i=-1,\text{odd}}^{2M}F_{2}(n_{i})F_{1}(n_{i-1})\;. \end{array}$$

Similarly, the probability that the present modality is 2 having observed the last 2*M* events is:

$$\begin{array}{l} \text{Pr}(M_{0}=2|N_{-2M:-1}=n_{-2M:-1})\\ \;=\prod_{i=-1,\text{odd}}^{2M}F_{1}(n_{i})F_{2}(n_{i-1})\;. \end{array}$$

We are better served by thinking about the normalized difference of the corresponding log likelihoods:

$$Q:=\frac{1}{2M}\text{log}\frac{P(M_{0}=1|N_{-2M:-1}=n_{-2M:-1})}{P(M_{0}=2|N_{-2M:-1}=n_{-2M:-1})}\;.$$

Some manipulation leads to:

$$Q=\frac{1}{2}(\frac{1}{M}\sum_{i=-1,\text{odd}}^{2M}\text{log}\frac{F_{2}(n_{i})}{F_{1}(n_{i})}+\frac{1}{M}\sum_{i=-1,\text{even}}^{2M}\text{log}\frac{F_{1}(n_{i})}{F_{2}(n_{i})}),$$

and, almost surely in the limit of *M* → ∞:

(A1) $$\frac{1}{M}\sum_{i=-1,\text{odd}}^{2M}\text{log}\frac{F_{1}(n_{i})}{F_{2}(n_{i})}\to \{\begin{array}{ll} D[F_{2}||F_{1}] & M_{0}=1\\ -D[F_{1}||F_{2}] & M_{0}=2 \end{array}\;,$$

where *D*[*P*||*Q*] is the information gain between *P* and *Q* (Cover and Thomas, 2006). And, we also have:

$$\frac{1}{M}\sum_{i=-1,\text{even}}^{2M}\text{log}\frac{F_{2}(n_{i})}{F_{1}(n_{i})}\to \{\begin{array}{ll} -D[F_{1}||F_{2}] & M_{0}=1\\ D[F_{2}||F_{1}] & M_{0}=2 \end{array}\;.$$

This implies that:

$$\underset{M\to \infty }{\text{lim}}Q=\frac{D[F_{2}||F_{1}]-D[F_{1}||F_{2}]}{2}\{\begin{array}{ll} 1 & M_{0}=1\\ -1 & M_{0}=2 \end{array}\;.$$

We only fail to identify the present modality almost surely from the semi-infinite past if ${\text{lim}}_{M\to \infty }Q=0$. Otherwise, the unnormalized difference of the log likelihoods:

$$\text{log}\frac{\text{Pr}(M_{0}=1|N_{:-1}=n_{:-1})}{\text{Pr}(M_{0}=2|N_{:-1}=n_{:-1})}$$

tends to ±∞, implying that one of the two probabilities has vanished. From the expression, $\lim_{M\to \infty }Q=0$ only happens when *D*[*F*_2_||*F*_1_] = *D*[*F*_1_||*F*_2_]. However, equality requires that *F*_1_(*n*) = *F*_2_(*n*) almost everywhere.

Given the present modality, we also need to know the counts since the last event in order to predict the future as well as possible. The proof of this is very similar to those given in Marzen and Crutchfield (2015). The conditional probability distribution of future given past is:

$$\text{Pr}(X_{0:}|X_{:0}=x_{:0})=\text{Pr}(N_{1:}|N_{0},X_{:0}=x_{:0})\text{Pr}(N_{0}|X_{:0}=x_{:0})\;.$$

Since the present modality is identifiable from the past *x*_:0_, and since interevent counts are independent given modality:

$$\text{Pr}(N_{1:}|N_{0},X_{:0}=x_{:0})=\text{Pr}(N_{1:}|M_{0}=m_{0}(n_{:-1}))\;.$$

So, it is necessary to know the modality in order to predict the future as well as possible. By virtue of how the alternating renewal process is generated, the second term is:

$$\text{Pr}(N_{0}|X_{:0}=x_{:0})=\text{Pr}(N_{0}|{N^{\prime }}_{0}={n^{\prime }}_{0},M_{0}=m_{0}(n_{:-1}))\;.$$

A very similar term was analyzed in Marzen and Crutchfield (2015), and that analysis revealed that it was necessary to store the counts since last spike when neither *F*_1_ nor *F*_2_ is eventually Δ-Poisson.

Identifying causal states S^+^ as the present modality M_0_ and the counts since last event $N_{0}^{\prime }$ immediately allows us to calculate the statistical complexity and entropy rate. The entropy rate can be calculated via:

$$\begin{array}{l} h_{\mu }=H[X_{0}|M_{0},N_{0}^{\prime }]\\ \;=\pi (M_{0}=1)H[X_{0}|M_{0}=1,N_{0}^{\prime }]\\ \;+\pi (M_{0}=2)H[X_{0}|M_{0}=2,N_{0}^{\prime }]. \end{array}$$

The statistical complexity is:

(A2) $$\begin{array}{l} C_{\mu }=H[S^{+}]\\ \;=H[M_{0},N_{0}^{\prime }]\\ \;=H[M_{0}]+\pi (M_{0}=1)H[N_{0}^{\prime }|M_{0}=1]\\ \;+\pi (M_{0}=2)H[N_{0}^{\prime }|M_{0}=2]\;. \end{array}$$

Finally, it is straightforward to show that the modality M_1_ at time step 1 and the counts to next event are the reverse-time causal states under the same conditions on *F*_1_ and *F*_2_. Therefore:

$$\begin{array}{l} E=I[S^{+};S^{-}]\\ \;=I[M_{0},N_{0}^{\prime };M_{1},N_{0}-N_{0}^{\prime }]\\ \;=I[M_{0};M_{1},N_{0}-N_{0}^{\prime }]\\ \;+I[N_{0}^{\prime };M_{1},N_{0}-N_{0}^{\prime }|M_{0}]\;. \end{array}$$

One can continue in this way to find formulae for other information measures of a discrete-time alternating renewal process.

These formulae can be rewritten terms of the modality-dependent information measures of Equations (13) and (14) if we recognize two things. First, the probability of a particular modality is proportional to the average amount of time spent in that modality. Second, for reasons similar to those outlined in Marzen and Crutchfield (2015), the probability of counts since last event given a particular present modality *i* is proportional to *w*_*i*_(*n*). Hence, in the infinitesimal time discretization limit, the probability of modality 1 is:

$$\pi (M_{0}=1)=\frac{\mu^{\left(1\right)}}{\mu^{\left(1\right)}+\mu^{\left(2\right)}}$$

and similarly for modality 2. Then, the entropy rate out of modality *i* is:

$$H[X_{1}|M_{0}=i,N_{0}^{\prime }]\sim \Delta t\left(\mu^{\left(i\right)}{\text{log}}_{2}\frac{1}{\Delta t}+h_{\mu }^{\left(i\right)}(\Delta t)\right)\;,$$

and the modality-dependent statistical complexity diverges as:

$$H[N_{0}^{\prime }|M_{0}=i]\sim {\text{log}}_{2}1/\Delta t+C_{\mu }(\Delta t)\;.$$

Finally, in continuous-time M_0_ and M_1_ limit to the same random variable, such that:

$$\underset{\Delta t\to 0}{\text{lim}}E(\Delta t)=H[M_{0}]+\underset{\Delta t\to 0}{\text{lim}}I[N_{0}^{\prime };N_{0}-N_{0}^{\prime }|M_{0}]\;.$$

Note that ${\text{E}}^{\left(i\right)}={\text{lim}}_{\Delta t\to 0}I\left[N_{0}^{\prime };N_{0}-N_{0}^{\prime }|M_{0}=i\right]$.

Bringing these results together, we substitute the above components into Equation (A2)'s expression for *C*_μ_ and, after details not shown here, find the expression quoted in the main text as Equation (16). Similarly, for *h*_μ_ and **E**, yielding the the formulae presented in the main text in Equations (15) and (17), respectively.

As a last task, as our hypothetical null model, we wish to find the information measures for the corresponding renewal process approximation. The ISI distribution of the alternating renewal process is:

(A3) $$\phi (t)=\frac{\mu^{\left(2\right)}\phi^{\left(1\right)}(t)+\mu^{\left(1\right)}\phi^{\left(2\right)}(t)}{\mu^{\left(1\right)}+\mu^{\left(2\right)}}$$

and its survival function is:

(A4) $$\Phi (t)=\frac{\mu^{\left(2\right)}\Phi^{\left(1\right)}(t)+\mu^{\left(1\right)}\Phi^{\left(2\right)}(t)}{\mu^{\left(1\right)}+\mu^{\left(2\right)}}\;.$$

Hence, its mean firing rate is:

(A5) $$\mu =\frac{1}{1/\mu^{\left(1\right)}+1/\mu^{\left(2\right)}}\;.$$

From Section 3, the entropy rate of the corresponding renewal process is:

$$\frac{h_{\mu }^{\text{ren}}(\Delta t)}{\Delta t}\sim \mu {\text{log}}_{2}\frac{1}{\Delta t}+\mu H[\phi (t)]\;;$$

compare Equation (15). And, the statistical complexity of the corresponding renewal process is:

$$C_{\mu }^{\text{ren}}(\Delta t)\sim {\text{log}}_{2}\frac{1}{\Delta t}+H[\mu \Phi (t)]\;.$$

The rate of divergence of $C_{\mu }^{\text{ren}}\left(\Delta t\right)$ is half the rate of divergence of the true *C*_μ_(Δ*t*), as given in Equation (16). Trivial manipulations, starting from $0\leq {\left(\frac{1}{\mu^{\left(1\right)}}-\frac{1}{\mu^{\left(2\right)}}\right)}^{2}$, imply that the rate of entropy-rate divergence is always less than or equal to the mean firing rate for an alternating renewal process. Jensen's inequality implies that each of the nondivergent components of these information measures for the renewal process is less than or equal to that of the alternating renewal process. The Data Processing Inequality (Cover and Thomas, 2006) also implies that the excess entropy calculated by assuming a renewal process is a lower bound on the true process' excess entropy.

## Appendix B

### Simplicity in complex neurons

Recall that our white noise-driven linear leaky integrate-and-fire (LIF) neuron has governing equation:

(A6) $$\dot{V}=b-V+a\eta (t)\;,$$

and, when *V* = 1, a spike is emitted and *V* is instantaneously reset to 0. We computed ISI survival functions from empirical histograms of 10^5^ ISIs. These ISIs were obtained by simulating Equation (A6) in Python/NumPy using an Euler integrator with time discretization of 1/1000 of log *b*/(*b*−1), which is the ISI in the noiseless limit.

The white noise-driven quadratic integrate-and-fire (QIF) neuron has governing equation:

(A7) $$\dot{V}=b+V^{2}+a\eta (t)\;,$$

and, when *V* = 100, a spike is emitted and *V* is instantaneously reset to −100. We computed ISI survival functions also from empirical histograms of trajectories with 10^5^ ISIs. These ISIs were obtained by simulating Equation (A7) in Python/NumPy using an Euler stochastic integrator with time discretization of 1/1000 of $\sqrt{\pi ∕b}$, which is the ISI in the noiseless limit when threshold and reset voltages are +∞ and −∞, respectively.

Figure 6 shows estimates of the following continuous-time information measures from this simulated data as they vary with mean firing rate μ and ISI coefficient of variation *C*_*V*_. This required us to estimate μ, *C*_*V*_, and:

$$C_{\mu }^{CT}:=\underset{\Delta t\to 0}{\text{lim}}(C_{\mu }(\Delta t)+{\text{log}}_{2}\Delta t),$$

$$E^{CT}:=\underset{\Delta t\to 0}{\text{lim}}E(\Delta t)\;,$$

$$h_{\mu }^{CT}:=\underset{\Delta t\to 0}{\text{lim}}\left(\frac{h_{\mu }(\Delta t)}{\Delta t}+\mu {\text{log}}_{2}\Delta t\right),\;\text{ and}$$

$$b_{\mu }^{CT}:=\underset{\Delta t\to 0}{\text{lim}}\frac{b_{\mu }(\Delta t)}{\Delta t}\;,$$

where the superscript *CT* is a reminder that these are appropriately regularized information measures in the continuous-time limit.

We estimated μ and *C*_*V*_ using the sample mean and sample coefficient of variation with sufficient samples so that error bars (based on studying errors as a function of data size) were negligible. The information measures required new estimators, however. From the formulae in Section 3, we see that:

(A8) $$C_{\mu }^{CT}={\text{log}}_{2}\frac{1}{\mu }-\mu \int_{0}^{\infty }\Phi (t){\text{log}}_{2}\Phi (t)dt\;,$$

(A9) $$\begin{array}{l} E^{CT}=\int_{0}^{\infty }\mu t\phi (t){\text{log}}_{2}(\mu \phi (t))dt\\ \;-2\int_{0}^{\infty }\mu \Phi (t){\text{log}}_{2}\Phi (t)dt\;, \end{array}$$

(A10) $$h_{\mu }^{CT}=-\mu \int_{0}^{\infty }\phi (t){\text{log}}_{2}\phi (t)\;,\;\text{ and}$$

(A11) $$\begin{array}{l} b_{\mu }^{CT}=-\mu (\int_{0}^{\infty }\phi (t)\int_{0}^{\infty }\phi (t^{\prime }){\text{log}}_{2}\phi (t+t^{\prime })dt^{\prime }dt\\ \;+\frac{1}{\text{log}2}-\int_{0}^{\infty }\phi (t){\text{log}}_{2}\phi (t)dt)\;. \end{array}$$

It is well known that the sample mean is a consistent estimator of the true mean, that the empirical cumulative density function is a consistent estimator of the true cumulative density function almost everywhere, and thus that the empirical ISI distribution is a consistent estimator of the true cumulative density function almost everywhere. In estimating the empirical cumulative density function, we introduced a cubic spline interpolator. This is still a consistent estimator as long as Φ(*t*) is three-times differentiable, which is the case for ISI distributions from integrate-and-fire neurons. We then have estimators of $C_{\mu }^{CT}$, **E**^*CT*^, $h_{\mu }^{CT}$, and $b_{\mu }^{CT}$ that are based on consistent estimators of μ, Φ(*t*), and ϕ(*t*) and that are likewise consistent.

We now discuss the finding evident in Figure 6, that the quantities ${\text{lim}}_{\Delta t\to 0}\text{E}\left(\Delta t\right)$ and ${\text{lim}}_{\Delta t\to 0}C_{\mu }+\log_{2}\left(\mu \Delta t\right)$ depend only on the ISI coefficient of variation *C*_*V*_ and not the mean firing rate μ. Presented in a different way, this is not so surprising. First, we use Marzen and Crutchfield (2015)'s expression for *C*_μ_ to rewrite:

$$\begin{array}{l} Q_{1}=\underset{\Delta t\to 0}{\text{lim}}\left(C_{\mu }(\Delta t)+{\text{log}}_{2}(\mu \Delta t)\right)\\ \;=-\mu \int_{0}^{\infty }\Phi (t){\text{log}}_{2}\Phi (t)dt \end{array}$$

and Equation (6) to rewrite:

$$\begin{array}{l} Q_{2}=\underset{\Delta t\to 0}{\text{lim}}E(\Delta t)\\ \;=2Q_{1}+\int_{0}^{\infty }\mu t\phi (t){\text{log}}_{2}(\mu \phi (t))dt\;. \end{array}$$

So, we only need to show that $-\mu \int_{0}^{\infty }\Phi \left(t\right)\log_{2}\Phi \left(t\right)dt$ and $\int_{0}^{\infty }\mu t\phi \left(t\right)\log_{2}\left(\mu \phi \left(t\right)\right)dt$ are independent of μ for two-parameter families of ISI distributions.

Consider a change of variables from *t* to *t*′ = μ*t*; then:

(A12) $$Q_{1}=-\int_{0}^{\infty }\Phi \left(t^{\prime }/\mu \right){\text{log}}_{2}(\Phi \left(t^{\prime }/\mu \right))dt^{\prime }$$

and

(A13) $$Q_{2}=2Q_{1}+\int_{0}^{\infty }t^{\prime }\phi \left(t^{\prime }/\mu \right){\text{log}}_{2}(\phi \left(t^{\prime }/\mu \right))dt^{\prime }\;.$$

For all of the ISI distributions considered here, $\phi \left(\frac{t^{\prime }}{\mu }\right)$ is still part of the same two-parameter family as ϕ(*t*), except that its mean firing rate is 1 rather than μ. Its *C*_*V*_ is unchanged. Hence, *Q*_1_ and *Q*_2_ are the same for a renewal process with mean firing rate 1 and μ, as long as the *C*_*V*_ is held constant. It follows that ${\text{lim}}_{\Delta t\to 0}\text{E}\left(\Delta t\right)$ and ${\text{lim}}_{\Delta t\to 0}C_{\mu }+\log_{2}\left(\mu \Delta t\right)$ are independent of μ and only depend on *C*_*V*_ for the two-parameter families of ISI distributions considered in Section 5. Similar arguments apply to understanding the universal *C*_*V*_-dependence of ${\text{lim}}_{\Delta t\to 0}b_{\mu }\left(\Delta t\right)∕\mu \Delta t$ and ${\text{lim}}_{\Delta t\to 0}h_{\mu }\left(\Delta t\right)∕\mu \Delta t+\log_{2}\left(\mu \Delta t\right)$.

In Figure 6, we also see that **E** seems to diverge as *C*_*V*_ → 0. Consider the following plausibility argument that suggests it diverges as log_2_1/*C*_*V*_ as *C*_*V*_ → 0. These two-parameter ISI distributions with finite mean firing rate μ and small *C*_*V*_ ≪ 1 can be approximated as Gaussians with mean 1/μ and standard deviation *C*_*V*_/μ. Recall from Equation (6) that we have:

$$\begin{array}{l} E=-2\int_{0}^{\infty }\mu \Phi (t){\text{log}}_{2}(\mu \Phi (t))dt\\ \;+\int_{0}^{\infty }\mu t\phi (t){\text{log}}_{2}(\mu \phi (t))dt\\ \;=-{\text{log}}_{2}\mu -2\mu \int_{0}^{\infty }\Phi (t){\text{log}}_{2}\Phi (t)dt\\ \;+\mu \int_{0}^{\infty }t\phi (t){\text{log}}_{2}\phi (t)dt\;. \end{array}$$

Note that as *C*_*V*_ → 0:

(A14) $$\Phi (t)\to \{\begin{array}{ll} 1 & t<\frac{1}{\mu }\\ \frac{1}{2} & t=\frac{1}{\mu }\\ 0 & t>\frac{1}{\mu } \end{array}$$

and so:

$$\underset{C_{V}\to 0}{\text{lim}}\int_{0}^{\infty }\Phi (t){\text{log}}_{2}\Phi (t)dt=0\;.$$

We assumed that for small *C*_*V*_, we can approximate:

$$\phi (t)\approx \frac{1}{\sqrt{2\pi C_{V}^{2}/\mu^{2}}}\text{exp}\left(-\frac{{\left(\mu t-1\right)}^{2}}{2C_{V}^{2}}\right)\;,$$

which then implies that:

(A15) $$\mu \int_{0}^{\infty }t\phi (t){\text{log}}_{2}\phi (t)dt\approx {\text{log}}_{2}\frac{\mu \sqrt{2\pi }}{C_{V}}-\frac{1}{2}.$$

So, for any ISI distribution tightly distributed about its mean ISI, we expect:

$$E\approx {\text{log}}_{2}\frac{1}{C_{V}}\;,$$

so that **E** diverges in this way. A similar asymptotic analysis also shows that as *C*_*V*_ → 0,

(A16) $$\underset{\Delta t\to 0}{\text{lim}}\frac{b_{\mu }(\Delta t)}{\Delta t}\approx \frac{1}{\text{log}2}(\frac{1}{2C_{V}^{2}}-\frac{1}{2})\;,$$

thereby explaining the divergence of $\lim_{\Delta t\to 0}b_{\mu }(\Delta t)/\Delta t$ evident in Figure 6.

Finally, a straightforward argument shows that *C*_μ_ is minimized at fixed μ when the neural spike train is periodic. We can rewrite *C*_μ_ in the infinitesimal time resolution limit as:

$$C_{\mu }(\Delta t)\sim {\text{log}}_{2}\left(\frac{1}{\mu \Delta t}\right)+\mu \int_{0}^{\infty }\Phi (t){\text{log}}_{2}\frac{1}{\Phi (t)}dt\;.$$

Note that 0 ≤ Φ (*t*) ≤ 1, and so $\int_{0}^{\infty }\Phi \left(t\right)\log_{2}\frac{1}{\Phi \left(t\right)}dt\geq 0$. We set it equal to zero by using the step function given in Equation (A14), which corresponds to a noiseless periodic process. So, the lower bound on *C*_μ_(Δ*t*) is log_2_1/μΔ*t*, and this bound is achieved by a periodic process.

## References

[B1] Abdallah S. A. Plumbley M. D. (2009). Information dynamics: patterns of expectation and surprise in the perception of music. Connect. Sci. 21, 89–117. 10.1080/09540090902733756

[B2] Abdallah S. A. Plumbley M. D. (2012). A measure of statistical complexity based on predictive information with application to finite spin systems. Phys. Lett. A. 376, 275–281. 10.1016/j.physleta.2011.10.066

[B3] Alivisatos A. P. Chun M. Church G. M. Greenspan R. J. Roukes M. L. Yuste R. (2012). The brain activity map project and the challenge of functional connectomics. Neuron 74, 970–974. 10.1016/j.neuron.2012.06.006 22726828 PMC3597383

[B4] Ara P. M. James R. G. Crutchfield J. P. (2015). The elusive present: hidden past and future dependence and why we build models. Available online at: http://arXiv.org:1507.00672 [cond-mat.stat-mech]. 10.1103/PhysRevE.93.022143 26986324

[B5] Archer E. Park I. M. Pillow J. W. (2012). Bayesian estimation of discrete entropy with mixtures of stick-breaking priors. Adv. Neural Info. Proc. Sys. 25, 2015–2023.

[B6] Atick J. J. (1992). Could information theory provide an ecological theory of sensory processing?, in Princeton Lectures on Biophysics, ed Bialek W. (Singapore: World Scientific), 223–289.

[B7] Averbeck B. B. Latham P. E. Pouget A. (2006). Neural correlations, population coding and computation. Nat. Rev. Neurosci. 7, 358–366. 10.1038/nrn1888 16760916

[B8] Barlow H. B. (1961). Possible principles underlying the transformation of sensory messages, in Sensory Communication, ed Rosenblith W. (Cambridge, MA: MIT Press), 217–234.

[B9] Bell A. J. Mainen Z. F. Tsodyks M. Sejnowski T. J. (1995). Balancing Conductances may Explain Irregular Cortical Firing. Technical Report, Institute for Neural Computation, San Diego.

[B10] Berry M. J. Warland D. K. Meister M. (1997). The structure and precision of retinal spike trains. Proc. Natl. Acad. Sci. U.S.A. 94, 5411–5416. 10.1073/pnas.94.10.5411 9144251 PMC24692

[B11] Bialek W. Nemenman I. Tishby N. (2001). Predictability, complexity, and learning. Neural Comp. 13, 2409–2463. 10.1162/089976601753195969 11674845

[B12] Bialek W. Ruderman D. L. Zee A. (1991). Optimal sampling of natural images: a design principle for the visual system? in Advances in Neural Information Processing 3, eds Lippman R. P. Moody J. E. Touretzky D. S. (San Mateo, CA: Morgan Kaufmann), 363–369.

[B13] Britten K. H. Newsome W. T. Shadlen M. N. Celebrini S. Movshon J. A. (1996). A relationship between behavioral choice and the visual responses of neurons in macaque MT. Vis. Neurosci. 13, 87–100. 10.1017/S095252380000715X 8730992

[B14] Butts D. A. Goldman M. S. (2006). Tuning curves, neuronal variability, and sensory coding. PLoS Biol. 4:e92. 10.1371/journal.pbio.0040092 16529529 PMC1403159

[B15] Cessac B. Cofre R. (2013). Spike train statistics and Gibbs distributions. Available online at: http://arXiv.org:1302.5007. 10.1016/j.jphysparis.2013.03.001 23501168

[B16] Costa M. Goldberger A. L. Peng C. K. (2002). Multiscale entropy analysis of complex physiologic time series. Phys. Rev. Lett. 89:068102. 10.1103/PhysRevLett.89.068102 12190613

[B17] Costa M. Goldberger A. L. Peng C. K. (2005). Multiscale entropy analysis of biological signals. Phys. Rev. E 71:021906. 10.1103/PhysRevE.71.021906 15783351

[B18] Cover T. M. Thomas J. A. (2006). Elements of Information Theory, 2nd Edn. New York, NY: Wiley-Interscience.

[B19] Crutchfield J. P. (1994). The calculi of emergence: Computation, dynamics, and induction. Physica D 75, 11–54. 10.1016/0167-2789(94)90273-9

[B20] Crutchfield J. P. Ellison C. J. Mahoney J. R. (2009). Time's barbed arrow: Irreversibility, crypticity, and stored information. Phys. Rev. Lett. 103:094101. 10.1103/PhysRevLett.103.094101 19792799

[B21] Crutchfield J. P. Feldman D. P. (2003). Regularities unseen, randomness observed: levels of entropy convergence. CHAOS 13, 25–54. 10.1063/1.1530990 12675408

[B22] Crutchfield J. P. Young K. (1989). Inferring statistical complexity. Phys. Rev. Let. 63, 105–108. 10.1103/PhysRevLett.63.105 10040781

[B23] Dan Y. Atick J. J. Reid R. C. (1996). Efficient coding of natural scenes in the lateral geniculate nucleus: experimental test of a computational theory. J. Neurosci. 16, 3351–3362. 8627371 10.1523/JNEUROSCI.16-10-03351.1996 PMC6579125

[B24] Destexhe A. Rudolph M. Paré D. (2003). The high-conductance state of neocortical neurons *in vivo*. Nat. Rev. Neurosci. 4, 739–751. 10.1038/nrn1198 12951566

[B25] Deweese M. (1996). Optimization principles for the neural code. Netw. Comp. Neural Sys. 7, 325–331. 10.1088/0954-898X/7/2/013 16754393

[B26] DeWeese M. R. Meister M. (1999). How to measure the information gained from one symbol. Network 10, 325–340. 10.1088/0954-898X/10/4/303 10695762

[B27] DeWeese M. R. Wehr M. Zador A. M. (2003). Binary spiking in auditory cortex. J. Neurosci. 23, 7940–7949. 12944525 10.1523/JNEUROSCI.23-21-07940.2003 PMC6740590

[B28] DeWeese M. R. Zador A. M. (2006). Non-Gaussian membrane potential dynamics imply sparse, synchronous activity in auditory cortex. J. Neurosci. 26, 12206–12218. 10.1523/JNEUROSCI.2813-06.2006 17122045 PMC6675435

[B29] Farmer J. D. Ott E. Yorke J. A. (1983). The dimension of chaotic attractors. Physica 7D, 153 10.1007/978-0-387-21830-4/11

[B30] Gao Y. Kontoyiannis I. Bienenstock E. (2008). Estimating the entropy of binary time series: methodology, some theory and a simulation study. Entropy 10, 71–99. 10.3390/entropy-e10020071

[B31] Gaspard P. Wang X.-J. (1993). Noise, chaos, and (ϵ, τ)-entropy per unit time. Phys. Rep. 235, 291–343. 10.1016/0370-1573(93)90012-3

[B32] Gerstner W. Kistler W. M. (2002). Spiking Neuron Models: Single Neurons, Populations, Plasticity. Cambridge, UK: Cambridge University Press.

[B33] Girardin V. (2005). On the different extensions of the ergodic theorem of information theory, in Recent Advances in Applied Probability Theory, eds Baeza-Yates R. Glaz J. Gzyl H. Husler J. Palacios J. L. (New York, NY: Springer), 163–179.

[B34] Haslinger R. Klinkner K. L. Shalizi C. R. (2010). The computational structure of spike trains. Neural Comp. 22, 121–157. 10.1162/neco.2009.12-07-678 19764880 PMC2849313

[B35] Jacobs A. L. Fridman G. Douglas R. M. Alam N. M. Latham P. E. Prusky G. T.. (2009). Ruling out and ruling in neural codes. Proc. Natl. Acad. Sci. U.S.A. 106, 5937–5941. 10.1073/pnas.0900573106 19297621 PMC2657589

[B36] James R. G. Burke K. Crutchfield J. P. (2014). Chaos forgets and remembers: measuring information creation, destruction, and storage. Phys. Lett. A 378, 2124–2127. 10.1016/j.physleta.2014.05.014

[B37] James R. G. Ellison C. J. Crutchfield J. P. (2011). Anatomy of a bit: information in a time series observation. CHAOS 21:037109. 10.1063/1.3637494 21974672

[B38] Koepsell K. Wang X. Hirsch J. A. Sommer F. T. (2010). Exploring the function of neural oscillations in early sensory systems. Front. Neurosci. 4, 53–61. 10.3389/neuro.01.010.2010 20582272 PMC2891629

[B39] Laughlin S. B. (1981). A simple coding procedure enhances a neuron's information capacity. Z. Naturforsch. 36c, 910–912. 7303823

[B40] Linsker R. (1989). An application of the principle of maximum information preservation to linear systems, in Advances in Neural Information Processing 1, ed Touretzky D. (San Mateo, CA: Morgan Kaufmann), 186–194.

[B41] London M. Roth A. Beeren L. Häusser M. Latham P. E. (2010). Sensitivity to perturbations *in vivo* implies high noise and suggests rate coding in cortex. Nature 466, 123–128. 10.1038/nature09086 20596024 PMC2898896

[B42] Mackay D. M. McCulloch W. W. (1952). The limiting information capacity of a neuronal link. Bull. Math. Biophys. 14, 127–135. 10.1007/BF02477711

[B43] Martius G. Der R. Ay N. (2013). Information driven self-organization of complex robotics behaviors. PLoS ONE 8:e63400. 10.1371/journal.pone.0063400 23723979 PMC3664628

[B44] Marzen S. Crutchfield J. P. (2014). Information anatomy of stochastic equilibria. Entropy 16, 4713–4748. 10.3390/e16094713

[B45] Marzen S. Crutchfield J. P. (2015). Informational and causal architecture of discrete-time renewal processes. Entropy 17, 4891–4917.

[B46] Mayer-Kress G. (ed.). (1986). Dimensions and Entropies in Chaotic Systems: Quantification of Complex Behavior. Berlin: Springer.

[B47] Meister M. Lagnado L. Baylor D. A. (1995). Concerted signaling by retinal ganglion cells. Science 270, 1207–1210. 10.1126/science.270.5239.1207 7502047

[B48] Nemenman I. Bialek W. de Ruyter van Steveninck R. R. (2004). Entropy and information in neural spike trains: progress on the sampling problem. Phys. Rev. E 69, 1–6. 10.1103/PhysRevE.69.056111 15244887

[B49] Nemenman I. Lewen G. D. Bialek W. de Ruyter van Steveninck R. R. (2008). Neural coding of natural stimuli: Information at sub-millisecond resolution. PLoS Comp. Bio. 4:e1000025. 10.1371/journal.pcbi.1000025 18369423 PMC2265477

[B50] Nirenberg S. Carcieri S. M. Jacobs A. L. Latham P. E. (2001). Retinal ganglion cells act largely as independent encoders. Nature 411, 698–701. 10.1038/35079612 11395773

[B51] Palmer S. E. Marre O. Berry II, M. J. Bialek W. (2013). Predictive information in a sensory population. Proc. Natl. Acad. Sci. U.S.A. 112, 6908–6913. 10.1073/pnas.1506855112 26038544 PMC4460449

[B52] Panzeri S. Treves A. Schultz S. Rolls E. T. (1999). On decoding the responses of a population of neurons from short time windows. Neural Comp. 11, 1553–1577. 10.1162/089976699300016142 10490938

[B53] Paz A. (1971). Introduction to Probabilistic Automata. New York, NY: Academic Press.

[B54] Rabiner L. R. (1989). A tutorial on hidden Markov models and selected applications. IEEE Proc. 77:257.

[B55] Rabiner L. R. Juang B. H. (1986). An introduction to hidden Markov models. IEEE ASSP Mag. 4–16. 10.1109/MASSP.1986.116534218428778

[B56] Reinagel P. Reid R. C. (2000). Temporal coding of visual information in the thalamus. J. Neurosci. 20, 5392–5400. 10884324 10.1523/JNEUROSCI.20-14-05392.2000 PMC6772338

[B57] Rieke F. Warland D. de Ruyter van Steveninck R. Bialek W. (1999). Spikes: Exploring the Neural Code. New York, NY: Bradford Book. 10.1126/science.2063199 2063199

[B58] Sakitt B. Barlow H. B. (1982). A model for the economical encoding of the visual image in cerebral cortex. Biol. Cybern. 43, 97–108. 10.1007/BF00336972 7059630

[B59] Schneidman E. Berry M. J. Segev R. Bialek W. (2006). Weak pairwise correlations imply strongly correlated network states in a neural population. Nature 440, 1007–1012. 10.1038/nature04701 16625187 PMC1785327

[B60] Schneidman E. Bialek W. Berry M. J. (2003). Synergy, redundancy, and independence in population codes. J. Neurosci. 23, 11539–11553. 14684857 10.1523/JNEUROSCI.23-37-11539.2003 PMC6740962

[B61] Shadlen M. N. Newsome W. T. (1995). Is there a signal in the noise? Curr. Opin. Neurobiol. 5, 248–250. 10.1016/0959-4388(95)80033-6 7620314

[B62] Shadlen M. N. Newsome W. T. (1998). The variable discharge of cortical neurons: Implications for connectivity, computation, and information coding. J. Neurosci. 18, 3870–3896. 9570816 10.1523/JNEUROSCI.18-10-03870.1998 PMC6793166

[B63] Shalizi C. R. Crutchfield J. P. (2001). Computational mechanics: Pattern and prediction, structure and simplicity. J. Stat. Phys. 104, 817–879. 10.1023/A:1010388907793

[B64] Shannon C. E. (1948). A mathematical theory of communication. Bell Sys. Tech. J. 27, 379–423, 623–656. 10.1002/j.1538-7305.1948.tb00917.x

[B65] Softky W. R. Koch C. (1993). The highly irregular firing of cortical cells is inconsistent with temporal integration of random EPSPs. J. Neurosci. 13, 334–350. 8423479 10.1523/JNEUROSCI.13-01-00334.1993 PMC6576320

[B66] Srinivasan M. V. Laughlin S. B. Dubs A. (1982). Predictive coding: A fresh view of inhibition in the retina. Proc. R. Soc. Lond. Ser. B 216, 427–459. 10.1098/rspb.1982.0085 6129637

[B67] Stein R. B. (1967). The information capacity of neurons using a frequency code. Biophys. J. 7, 797–826. 10.1016/S0006-3495(67)86623-2 19210999 PMC1368193

[B68] Stevens C. F. Zador A. (1996). Information through a spiking neuron, in Advance Neural Information Processing System, eds Touretzky D. Mozer M. C. Hasselmo M. E. (Cambridge, MA: MIT Press), 75–81.

[B69] Stevens C. F. Zador A. M. (1998). Input synchrony and the irregular firing of cortical neurons. Nat. Neurosci. 1, 210–217. 10.1038/659 10195145

[B70] Strong S. P. Koberle R. de Ruyter van Steveninck R. Bialek W. (1998). Entropy and information in neural spike trains. Phys. Rev. Lett. 80, 197–200. 10.1103/PhysRevLett.80.197

[B71] Tchernookov M. Nemenman I. (2013). Predictive information in a nonequilibrium critical model. J. Stat. Phys. 153, 442–459. 10.1007/s10955-013-0833-6

[B72] Theunissen F. E. Miller J. P. (1991). Representation of sensory information in the cricket cercal sensory system. ii: information theoretic calculation of system accuracy and optimal tuning curve widths of four primary interneurons. J. Neurophys. 66, 1690–1703. 1765802 10.1152/jn.1991.66.5.1690

[B73] Tokdar S. Xi P. Kelly R. C. Kass R. E. (2010). Detection of bursts in extracellular spike trains using hidden semi-Markov point process models. J. Comput. Neurosci. 29, 203–212. 10.1007/s10827-009-0182-2 19697116

[B74] Treves A. Panzeri S. (1995). The upward bias in measures of information derived from limited data samples. Neural Comp. 7, 399–407. 10.1162/neco.1995.7.2.399

[B75] Verdú S. Weissman T. (2006). Erasure entropy, in *IEEE International Symposium on Information Theory (ISIT 2006)* (Seattle, WA), 98–102. 10.1109/ISIT.2006.261682

[B76] Victor J. D. (2002). Binless strategies for estimation of information from neural data. Phys. Rev. E 66:051903. 10.1103/PhysRevE.66.051903 12513519

[B77] Vilela R. D. Lindner B. (2009). Are the input parameters of white noise driven integrate and fire neurons uniquely determined by rate and CV? J. Theo. Bio. 257, 90–99. 10.1016/j.jtbi.2008.11.004 19063904

[B78] Yang Y. Zador A. M. (2012). Differences in sensitivity to neural timing among cortical areas. J. Neurosci. 32, 15142–15147. 10.1523/JNEUROSCI.1411-12.2012 23100435 PMC3506386

[B79] Yeung R. W. (2008). Information Theory and Network Coding. New York, NY: Springer.

